# Dietary profiling of physical frailty in older age phenotypes using a machine learning approach: the Salus in Apulia Study

**DOI:** 10.1007/s00394-022-03066-9

**Published:** 2022-12-09

**Authors:** Sara De Nucci, Roberta Zupo, Rossella Donghia, Fabio Castellana, Domenico Lofù, Simona Aresta, Vito Guerra, Ilaria Bortone, Luisa Lampignano, Giovanni De Pergola, Madia Lozupone, Rossella Tatoli, Giancarlo Sborgia, Sarah Tirelli, Francesco Panza, Tommaso Di Noia, Rodolfo Sardone

**Affiliations:** 1 Unit of Data Sciences and Technology Innovation for Population Health, National Institute of Gastroenterology “Saverio de Bellis,” Research Hospital, Castellana Grotte, Bari, Italy; 2 Department of Electrical and Information Engineering, Polytechnic of Bari, Bari, Italy; 3 Unit of Geriatrics and Internal Medicine, National Institute of Gastroenterology “Saverio de Bellis,” Research Hospital, Castellana Grotte, Bari, Italy; 4 Department of Basic Medical Sciences, Neuroscience and Sense Organs, University of Bari “Aldo Moro”, Bari, Italy

**Keywords:** Food intake, Dietary habits, Mediterranean diet, Salus in Apulia Study, Older population, Physical frailty

## Abstract

**Purpose:**

Growing awareness of the biological and clinical value of nutrition in frailty settings calls for further efforts to investigate dietary gaps to act sooner to achieve focused management of aging populations. We cross-sectionally examined the eating habits of an older Mediterranean population to profile dietary features most associated with physical frailty.

**Methods:**

Clinical and physical examination, routine biomarkers, medical history, and anthropometry were analyzed in 1502 older adults (65 +). CHS criteria were applied to classify physical frailty, and a validated Food Frequency Questionnaire to assess diet. The population was subdivided by physical frailty status (frail or non-frail). Raw and adjusted logistic regression models were applied to three clusters of dietary variables (food groups, macronutrients, and micronutrients), previously selected by a LASSO approach to better predict diet-related frailty determinants.

**Results:**

A lower consumption of wine (OR 0.998, 95% CI 0.997–0.999) and coffee (OR 0.994, 95% CI 0.989–0.999), as well as a cluster of macro and micronutrients led by PUFAs (OR 0.939, 95% CI 0.896–0.991), zinc (OR 0.977, 95% CI 0.952–0.998), and coumarins (OR 0.631, 95% CI 0.431–0.971), was predictive of non-frailty, but higher legumes intake (OR 1.005, 95%CI 1.000–1.009) of physical frailty, regardless of age, gender, and education level.

**Conclusions:**

Higher consumption of coffee and wine, as well as PUFAs, zinc, and coumarins, as opposed to legumes, may work well in protecting against a physical frailty profile of aging in a Mediterranean setting. Longitudinal investigations are needed to better understand the causal potential of diet as a modifiable contributor to frailty during aging.

**Supplementary Information:**

The online version contains supplementary material available at 10.1007/s00394-022-03066-9.

## Introduction

Biodemographics indicate a fast-growing and aging world population. Life expectancy at age 65 has increased in nearly every country over the past four decades [1]. Looking closer, European projections suggest over 36% of the population will be aged over 65 by 2050 [2]. Such a shift imposes severe burdens on medical care and social security systems due to multiple chronic illnesses and disabilities. Current research efforts in managing health risks in later life are heavily focused on the functional physiological decline during aging that makes older adults more vulnerable to external stressors. Paths explaining this decline involve multiple biological dimensions, hitherto defined by several constructs [3] but better clarified by a one-dimensional physical model composed of interconnected domains [4]. The insidious subclinical course of this multi-level functional impairment results in the onset of a physical frailty phenotype that slowly brings older people closer to loss of independence, disability, malnutrition, multimorbidity, and death [5–9].

Nutrition plays a central role in the multifactorial etiology of physical frailty, covering more than two-thirds of existing frailty concepts [7]. Taking preventive action on dietary management in older adults has successfully proven to curb health risk trajectories in this population, and survival of frail individuals suggests that preventive nutritional management can successfully reduce key adverse health outcomes [10]. From this preventative mindset, we recently outlined a series of nutritional imbalance conditions (i.e., low body mass index, low skeletal muscle index, higher daily sodium intake, and lower daily potassium and iron intake) that, taken together, accounted for a doubled risk of overall mortality in our frail population [11]. This novel algorithm outlining a nutritional frailty phenotype featured both anthropometric and dietary arms. However, while there is solid scientific consistency for anthropometric determinants, there are still gaps regarding foods and dietary foods and patterns implicated in accelerating risk trajectories. The combination of unfavorable physiological conditions such as reduced appetite and thirst, poor oral health, multimorbidity, disability, and social deprivation inevitably leads to gradual changes in eating habits that ultimately result in the nutritional imbalances typical of aging. Since diet is a modifiable health risk driver, nutrition is rapidly becoming an active focus in health promotion efforts in the field of multidimensional aging management.

At the current state of evidence, research on the link between diet and frailty is based primarily on the investigation of overall diet quality [12, 13], food groups [14–17], dietary patterns [18–20], and a priori indices [12, 21]. Much emphasis has been placed on the Mediterranean lifestyle as a healthy approach to preventing the risk of physical frailty, as reported by some reports on the elderly population [22]. In clinical intervention trials, instead, there is some emphasis on the effect of protein supplementation [23], given the well-established contribution of protein malnourishment to muscle wasting underlying physical decline during aging [24, 25].

As food group recommendations, rather than recommended nutrient intakes, are used as a national guide to healthy eating, targeting specific food groups might be helpful to better track the risk trajectories of nutritional frailty. Promoting dietary health by means of specific food groups as part of educational interventions rather than recommending nutrients intake might be a more coherent approach for older adults, given the prevalence of cognitive decline and literacy issues in this population setting [26]. In this regard, prospective Spanish data have shown the consumption of ultra-processed foods to be strongly associated with the frailty risk in older adults [27]. Also, long-term overconsumption of added sugars has demonstrated a negative association [28], and there is some evidence that a greater consumption of fish, white meat, fruits, and vegetables acts against the onset of frailty, although much remains to be elucidated in this context [29]. On this basis, we used data from the Salus in Apulia population-based study of Southern Italy to investigate foods and nutrients more predictive of physical frailty, using a novel machine learning selection approach: the LASSO (least absolute shrinkage and selection operator). LASSO is a methodological approach to define the best model in terms of goodness of fit and therefore to select variables that better explain the outcome avoiding overfitting [30]. It is definitely the best choice when you have to select many variables and avoid putting them all in the model and increasing the overfitting problem [31, 32]. However, the variables are not automatically associated from a statistical point of view, which is why the coefficients of each individual variable must be interpreted. The choice to apply this machine learning method to a Mediterranean population-based setting represents a novel aspect with respect to the topic diet and frailty.

## Methods

### Study population

Participants were recruited from the electoral rolls of Castellana Grotte (Apulia, Southern Italy). The sampling framework was the list of the health registry office until 31st December 2011, which included 19,675 subjects, 3981 aged 65 + years. All subjects aged 65 + on 31st December 2011 were invited to participate (*n* = 3981) in the “Salus in Apulia Study” [33], a cohort study conducted at the National Institute of Gastroenterology IRCCS “Saverio De Bellis” Research Hospital. Of the whole sample, only 1502 older subjects underwent all the assessments and were eligible for inclusion in this study. All participants were enrolled from January 2012 to January 2019. All participants signed informed consent before their examination, and the study was approved by the Institutional Review Board (IRB) of the head institution, the National Institute of Gastroenterology and Research Hospital “S. de Bellis” in Castellana Grotte, Bari, Italy (Protocol n. 68/2019). The study met the principles of the Helsinki Declaration and adhered to the “Standards for Reporting Diagnostic Accuracy Studies” (STARD) guidelines (http://www.stard-statement.org/) and the “Strengthening the Reporting of Observational Studies in Epidemiology” (STROBE) guidelines.

### Sociodemographic and clinical assessment

Education was defined by years of schooling. Smoking status was assessed with the single question, “Are you a current smoker?”. Extemporaneous ambulatory systolic blood pressure (SBP) and diastolic blood pressure (DBP) were determined in a sitting position after at least a 10-min rest and at least three different times, using the OMRON M6 automatic blood pressure monitor. A blood sample was collected in the morning after overnight fasting to measure the levels of fasting blood glucose (FBG), glycated hemoglobin (HbA1c), total cholesterol, high-density lipoprotein (HDL) cholesterol, and low-density lipoprotein (LDL) cholesterol and triglycerides, using standard automated enzymatic colorimetric methods (AutoMate 2550, Beckmann Coulter, Brea, Ca, US) under strict quality control. LDL cholesterol was calculated using the Friedewald equation. Plasma glucose was determined using the glucose oxidase method (Sclavus, Siena, Italy). Blood cell count was determined by a Coulter Hematology analyzer (Beckman–Coulter, Brea, CA). Serum high-sensitivity C-reactive protein (CRP) was assayed using a latex particle-enhanced immunoturbidimetric assay (Kamiya Biomedical Company, Seattle, WA) (reference range: 0–5.5 mg/L; interassay coefficient of variation: 4.5%). Serum interleukin (IL)-6 and tumor growth factor- (TNF-α) were assayed using the quantitative sandwich enzyme technique ELISA (QuantiKine High Sensitivity Kit, R&D Systems, Minneapolis, MN, and QuantiGlo immunoassay from R&D Systems, Minneapolis, MN). Interassay coefficients of variation were 11.7% for IL-6 and 13.0% for TNFα. Inflammatory marker assays were analyzed at the same laboratory, applying strict quality control procedures. Multimorbidity status was defined as the co-presence of two or more chronic diseases [diabetes mellitus, hypertension, dyslipidemia, peripheral age-related hearing loss (ARHL), vision loss, cognitive impairment, asthma, and chronic obstructive pulmonary disease], as described elsewhere [5].

### Anthropometry and diet assessment

Anthropometric and dietary assessment procedures were performed under the supervision of a senior nutritionist (RZ). Height and weight were measured using a Seca 220 altimeter and a Seca 711 scale. Body mass index (BMI) was calculated as kg/m^2^. Diet was assessed with a self-administered Food Frequency Questionnaire, previously validated in our community [34], to investigate dietary habits over the previous year, as described in detail elsewhere [33]. Briefly, it is a questionnaire structured in eleven sections that partly mirror the sequence of food intake throughout the day and includes queries on weekly intake frequency of foods such as grains, meat, fish, milk and dairy products, vegetables, legumes, fruits, miscellaneous foods, water and alcoholic beverages, olive oil and other edible fats, coffee/sugar, and salt. Supplementary Table S1 shows the concordance of the single foods in the questionnaire and the food grouping used in the analyses. Total energy, macronutrients, micronutrients, and polyphenols intake were calculated using reference tables based on the Agricultural Research Council (CRA) [35], Food Composition Database for Epidemiological Studies in Italy (BDA), and Phenol-Explorer [36]. Nutrient quantity was calculated per 100 g of consumption of macronutrients, micronutrients, and polyphenols.

### Physical activity and physical frailty assessment

According to a binary cutoff validated value, subjects were categorized as physically inactive\sedentary or physically active [37]. Assessment of the physical frailty status was performed using CHS criteria by Fried, slightly modified for the present study, namely positivity to three or more of the following: weight loss, exhaustion, low levels of physical activity, weakness, and slow gait, as detailed elsewhere [5]. The 5-repetition sit-to-stand test, a measure of the amount of time it takes a patient to get up 5 times from a sitting position without using their arms, was used as a metric of weakness, using > 15 s as the diagnostic threshold [38]. The nutritional status was assessed with the Mini Nutritional Assessment, which provides weight loss and nutritional intake information, using a threshold score of < 23.5 [39]. Gait speed was assessed within our gait analysis laboratory using a 5-m walk test and classified as slow if the recorded time is greater than or equal to the cutoff point of 0.6 m/s as the slow gait speed. Physical activity was assessed through a questionnaire administered by an interviewer [40]. Specifically, subjects were asked to indicate their average level of physical activity during the previous year, choosing from 6 response categories (0–5), including duration, frequency, and intensity of physical activity. We used variable as a dichotomous with cutoff value < 2, based on the results of a recent study of a subset of our population that examined the relationship between activity [41] energy expenditure estimated by wrist accelerometers and self-reported physical activity intensity (InCHIANTI structured interview questionnaire) [37]. The gait test was used to measure exhaustion and assessed using a modified version of the Berg Stool-Stepping task [41]. Finally, the entire sample was assigned to two different groups based on the number of physical frailty components. Subjects meeting ≥ 3 criteria were included in the frailty group, and all the others in the non-frailty group.

### Advanced statistical analysis

The whole sample was subdivided according to the physical frailty phenotype condition (yes/no) to assess differences in terms of frequency and associations with clinical, sociodemographic, and dietary variables. Normal distributions of quantitative variables were tested using the Shapiro–Wilk test. Data were reported as Mean ± Standard Deviations (M ± SD) for continuous measures and frequency and percentages (%) for all categorical variables. A statistical approach based on the null hypothesis significance test (NHST) was not used to focus on the practical differences between the groups in terms of effect size; instead, significant differences in the magnitude of association, i.e., effect size (ES), were calculated and used to assess the prevalence of physical frailty condition groups (frailty/ non-frailty) and other categorical variables and their 95% CIs. Differences between continuous variables were calculated using Cohen’s *d* difference between means, Hedge’s *g* when the assumption of a similar variance was violated, and their ES using the confidence intervals [42]. ES is a quantitative measure of the magnitude of the effect. The larger the effect size, the stronger the relationship between the two variables. Cohen suggested that *d* = 0.2 be considered a “small” effect size, 0.5 represents a “medium” effect size, and 0.8 a “large” effect size [43].

A least absolute shrinkage and selection operator (LASSO) variable selection method was applied to determine the most relevant predictors in terms of food groups, macronutrients, and micronutrients [30]. The logistic LASSO model is a shrinkage method that can actively select from a large and potentially multicollinear set of variables in the regression, resulting in a more relevant and interpretable set of predictors. LASSO was run based on minimizing the regression coefficients to reduce the probability of overfitting, producing the coefficient equal to 0, and then selecting non-zero variables that should remain in the model. To set variables, LASSO was run on the training dataset. A Lambda penalty parameter (*λ*) was identified by LASSO using cross-validation. This penalty was the sum of the absolute values of the coefficients. LASSO restricted the coefficient estimates toward zero by setting the variables exactly equal to zero when *λ* was large enough. As *λ* was small, the result was essentially the least squares estimates. As *λ* increased, shrinkage occurred, allowing the variables at zero to be thrown out. Variables selected by LASSO from each of the three clusters of dietary variables, i.e., food groups, micronutrients, and macronutrients, were fitted into two hierarchical nested models and adjusted multiple logistic regression models were applied to identify the direction and effect size of the association with the physical frailty condition. Risk estimators were reported as Odds Ratios (OR) and 95% Confidence Intervals (95% CIs).

The methodological approach design and statistical analyses were managed by a senior epidemiologist (RS), a biostatistician (RD) and artificial intelligence experts (D.L. and T.D.) using StataCorp. 2021. Stata Statistical Software: Release 17. College Station, TX: StataCorp LLC.

## Results

The average age of the examined population (*n* = 1502) was 73.4 ± 6.3 years and slightly dominated by males (53.5%, *n* = 749). Table 1 summarizes the main differences in clinical, sociodemographic, and dietary variables according to the physical frailty condition (frailty or non-frailty). Females were more affected by physical frailty concerns (Effect Size 0.02, 95% CI 0.01–0.16), accounting for 13.5% overall (*n* = 204), and older age (ES − 0.26, 95% CI − 0.40 to − 0.11) and lower education level (ES 0.16, 95% CI 0.02–0.31) were both hallmarks of our frail population. This condition was found to be closely associated with multimorbidity (ES 0.003, 95% CI 0.04–0.18), with a higher burden of age-related hearing loss (ARHL) (ES 0.04, 95% CI 0.004–0.14), cognitive impairment (ES 0.23, 95% CI 0.08–0.38), and diabetes (ES 0.005, 95% CI 0.02–0.14). Consistently, descriptive analysis of fluid biomarkers showed much higher average serum HbA1c levels among frail individuals (ES − 0.18, 95% CI − 0.33 to − 0.03). Descriptive data on eating habits by food group indicated lower consumption of coffee (ES 0.22, 95% CI 0.07–0.37), wine (ES 0.26, 95% CI 0.12–0.41), and liquor (ES 0.17, 95% CI 0.02–0.31) among frail compared with non-frail subjects. Considering macro and micronutrients, lower consumption of alcohol (ES 0.28, 95% CI 0.13–0.43), dihydroflavonols (ES 0.26, 95% CI 0.12–0.41), stilbenes (ES 0.27, 95% CI 0.12–0.42), hydroxybenzaldehydes (ES 0.27, 95% CI 0.12–0.42), hydroxycoumarins (ES 0.27, 95% CI 0.12–0.42), and naphthoquinones (ES 0.17, 95% CI 0.02–0.31) emerged in the frailty versus non-frailty group.

**Table 1:** Sociodemographic, clinical and nutritional variables in patients with physical and cognitive frailty

Parameters^*^	Physical frailty		
	No (*n* = *1195*)	Yes (*n* = *204*)	Effect’s size (95%)^ψ^
Sociodemographic and clinical variables			
Sex (F) (%)	540 (45.19)	110 (53.92)	0.02 (0.01 to 0.16)
Age (years)	73.19 ± 6.25	74.80 ± 6.41	− 0.26 (− 0.40 to − 0.11)
Smoking (%)	103 (8.62)	11 (5.39)	0.07 (− 0.07 to 0.002)
Education (years)	7.09 ± 3.74	6.46 ± 4.09	0.16 (0.02 to 0.31)
BMI (Kg/m^2^)	28.40 ± 4.81	28.98 ± 5.31	− 0.12 (− 0.27 to 0.03)
Multimorbidity (≥ 2) (%)	530 (44.35)	113 (55.39)	0.003 (0.04 to 0.18)
Diabetes mellitus (%)	145 (12.13)	42 (20.59)	0.005 (0.02 to 0.14)
Hypertension (%)	845 (70.71)	151 (74.02)	0.32 (− 0.03 to 0.10)
Dyslipidemia (%)	7 (0.59)	0.00 (0.00)	–
ARHL (%)	250 (20.92)	57 (27.94)	0.04 (0.004 to 0.14)
COPD/BPCO (%)	212 (17.74)	45 (22.06)	0.16 (− 0.02 to 0.10)
Vision loss (%)	43 (3.60)	7 (3.43)	0.90 (− 0.03 to 0.02)
Asthma (%)	109 (9.12)	22 (10.78)	0.47 (− 0.03 to 0.06)
LLD (%)	135 (11.30)	25 (12.25)	0.70 (− 0.04 to 0.06)
Systolic BP (mmHg)	133.04 ± 14.33	134.90 ± 14.90	− 0.13 (− 0.28 to 0.02)
Diastolic BP (mmHg)	78.38 ± 7.78	78.14 ± 7.89	0.03 (− 0.12 to 0.18)
Total cholesterol (mg/dL)	183.87 ± 36.80	180.86 ± 40.46	0.08 (− 0.07 to 0.23)
HDL cholesterol (mg/dL)	48.80 ± 13.11	47.01 ± 11.69	0.14 (− 0.10 to 0.29)
LDL cholesterol (mg/dL)	113.23 ± 31.10	111.48 ± 34.53	0.05 (− 0.09 to 0.20)
Triglycerides (mg/dL)	105.70 ± 59.16	108.32 ± 58.68	− 0.04 (− 0.19 to 0.10)
Hb (g/dL)	13.85 ± 1.47	13.67 ± 1.57	0.12 (− 0.03 to 0.26)
HbA1c (mmol/mol)	40.25 ± 10.41	42.12 ± 11.06	− 0.18 (− 0.33 to − 0.03)
Interleukin-6 (pg/mL)	3.92 ± 6.88	3.93 ± 5.55	− 0.001 (− 0.15 to 0.15)
TNF-α (µg/mL)	2.83 ± 3.99	2.73 ± 2.02	0.03 (− 0.12 to 0.17)
Red blood cells (10^6^/µL)	4.82 ± 1.16	4.77 ± 0.56	0.04 (− 0.10 to 0.19)
Platelets (10^3^/µL)	220.80 ± 56.25	219.03 ± 64.75	0.03 (− 0.12 to 0.18)
White blood cells (10^3^/µL)	6.09 ± 1.80	6.31 ± 2.38	− 0.12 (− 0.27 to 0.03)
C-Reactive Protein (mg/dL)	0.59 ± 0.86	0.63 ± 0.98	− 0.05 (− 0.19 to 0.10)
25-Hydroxyvitamin D3 (nmol/L)	39.15 ± 17.72	40.23 ± 17.31	− 0.06 (− 0.21 to 0.09)
Weight loss	58 (4.85)	29 (14.22)	< 0.001 (0.04 to 0.14)
Weakness	245 (20.50)	191 (93.63)	< 0.001 (0.69 to 0.77)
Exhaustion	48 (4.02)	112 (54.90)	< 0.001 (0.44 to 0.58)
Slowness	151 (12.64)	188 (92.16)	< 0.001 (0.75 to 0.84)
Low physical activity	202 (16.90)	170 (83.33)	< 0.001 (0.61 to 0.72)
MMSE	26.74 ± 3.95	25.81 ± 3.99	0.23 (0.08 to 0.38)
Food groups^¥^			
Dairy	102.60 ± 107.30	118.26 ± 121.69	− 0.14 (− 0.29 to 0.005)
Low fat dairy	100.53 ± 107.82	106.13 ± 110.66	− 0.05 (− 0.20 to 0.10)
Eggs	8.18 ± 9.04	8.37 ± 9.18	− 0.02 (− 0.17 to 0.13)
White meat	26.49 ± 37.78	27.03 ± 33.75	− 0.01 (− 0.16 to 0.13)
Red meat	23.40 ± 27.26	21.15 ± 19.28	0.08 (− 0.06 to 0.23)
Processed meat	15.55 ± 21.20	14.77 ± 17.26	0.04 (− 0.11 to 0.19)
Fish	26.66 ± 45.20	24.35 ± 25.52	0.05 (− 0.9 to 0.20)
Seafood/Shellfish	10.18 ± 27.89	10.11 ± 19.20	0.003 (− 0.15 to 0.15)
Leafy vegetables	59.48 ± 66.46	62.37 ± 62.17	− 0.04 (− 0.19 to 0.10)
Fruiting vegetables	95.29 ± 83.78	95.52 ± 76.64	− 0.003 (− 0.15 to 0.14)
Root vegetables	12.31 ± 29.07	11.09 ± 18.25	0.04 (− 0.10 to 0.19)
Other vegetables	81.98 ± 82.58	82.74 ± 76.56	− 0.01 (− 0.16 to 0.14)
Legumes	37.36 ± 29.43	43.42 ± 38.38	− 0.20 (− 0.34 to 0.05)
Potatoes	13.35 ± 19.36	13.72 ± 16.93	− 0.02 (− 0.17 to 0.13)
Fruits	618.32 ± 523.65	611.36 ± 571.92	0.01 (− 0.13 to 0.16)
Nuts	7.38 ± 16.03	6.70 ± 14.19	0.04 (− 0.10 to 0.19)
Grains	156.00 ± 107.61	156.08 ± 105.58	− 0.001 (− 0.15 to 0.15)
Olives and vegetable oil	51.91 ± 36.91	52.08 ± 41.06	− 0.004 (− 0.15 to 0.14)
Sweets	23.21 ± 36.24	20.26 ± 20.95	0.08 (− 0.06 to 0.23)
Sugary	10.46 ± 16.73	11.16 ± 38.05	− 0.03 (− 0.18 to 0.11)
Juices	6.58 ± 20.61	7.21 ± 21.46	− 0.03 (− 0.18 to 0.12)
Caloric drinks	9.07 ± 52.83	5.76 ± 51.65	0.06 (− 0.08 to 0.21)
Ready to eat dish	33.57 ± 49.43	32.39 ± 31.48	0.02 (− 0.12 to 0.17)
Coffee	47.89 ± 30.10	41.32 ± 27.59	0.22 (0.07 to 0.37)
Wine	128.63 ± 168.21	85.24 ± 128.46	0.26 (0.12 to 0.41)
Beer	20.72 ± 75.32	12.66 ± 55.06	0.11 (− 0.04 to 0.26)
Spirits	1.65 ± 5.79	0.73 ± 2.67	0.17 (0.02 to 0.31)
Water	654.51 ± 297.29	696.16 ± 315.37	− 0.14 (− 0.29 to 0.01)
Macronutrients^¥^			
H_2_O	1877.78 ± 734.18	1879.02 ± 735.74	− 0.002 (− 0.15 to 0.15)
Energy (Kcal)	1762.15 ± 773.33	1736.36 ± 740.35	0.03 (− 0.11 to 0.18)
Carbohydrates available	231.31 ± 107.34	230.76 ± 108.47	0.005 (− 0.14 to 0.15)
Starch	114.67 ± 64.29	117.37 ± 64.72	− 0.04 (− 0.19 to 0.11)
Carbohydrates soluble	104.51 ± 60.62	100.55 ± 65.83	0.06 (− 0.08 to 0.21)
Total fiber	27.56 ± 15.49	27.80 ± 16.16	− 0.01 (− 0.16 to 0.13)
Soluble fiber	6.61 ± 4.32	6.63 ± 4.71	− 0.005 (− 0.15 to 0.14)
Insoluble fiber	16.38 ± 10.27	15.60 ± 10.41	− 0.02 (− 0.17 to 0.13)
Proteins	77.03 ± 41.06	78.78 ± 36.81	− 0.04 (− 0.19 to 0.10)
Lipids	46.94 ± 27.37	47.15 ± 27.74	− 0.01 (− 0.16 to 0.14)
Cholesterol	170.92 ± 130.32	172.61 ± 104.87	− 0.01 (− 0.16 to 0.13)
Saturated fatty acids	36.86 ± 18.40	34.94 ± 18.69	0.10 (− 0.04 to 0.25)
Palmitic acid	24.94 ± 12.44	23.57 ± 12.46	0.11 (− 0.04 to 0.26)
Stearic acid	6.33 ± 3.17	6.12 ± 3.28	0.06 (− 0.08 to 0.21)
Monounsatured fatty acids	20.46 ± 12.21	19.88 ± 11.02	0.05 (− 0.10 to 0.20)
Oleic acid	18.34 ± 11.22	17.79 ± 10.04	0.05 (− 0.10 to 0.20)
Palmitoleic acid	0.92 ± 0.77	0.94 ± 0.63	− 0.02 (− 0.17 to 0.12)
Polyunsatured fatty acids	26.40 ± 14.88	23.12 ± 13.85	0.22 (0.07 to 0.37)
EPA	0.11 ± 0.24	0.10 ± 0.11	0.04 (− 0.11 to 0.19)
DHA	0.14 ± 0.29	0.13 ± 0.15	0.05 (− 0.10 to 0.20)
Alcohol	14.44 ± 18.61	9.42 ± 14.10	0.28 (0.13 to 0.43)
Micronutrients^¥^			
Na	1577.29 ± 979.73	1609.89 ± 838.58	− 0.03 (− 0.18 to 0.11)
K	4215.06 ± 1948.85	4088.50 ± 1881.74	0.06 (− 0.08 to 0.21)
Ca	992.77 ± 510.35	1056.91 ± 592.43	− 0.12 (− 0.27 to 0.02)
P	1342.72 ± 645.08	1359.70 ± 661.75	− 0.03 (− 0.17 to 0.12)
Mg	316.67 ± 133.52	302.55 ± 124.49	0.11 (− 0.04 to 0.25)
Fe	13.27 ± 6.11	12.68 ± 5.47	0.10 (− 0.05 to 0.25)
Cu	1.68 ± 1.12	1.60 ± 0.81	0.08 (− 0.07 to 0.23)
Zn	59.43 ± 34.20	52.17 ± 32.14	0.21 (0.06 to 0.36)
Se	48.49 ± 47.74	47.79 ± 24.18	0.01 (− 0.13 to 0.16)
Mn	19.75 ± 15.73	19.28 ± 14.84	0.03 (− 0.12 to 0.18)
Vitamin A	1234.09 ± 1871.59	1198.33 ± 818.01	0.02 (− 0.13 to 0.17)
Vitamin D	2.75 ± 3.34	2.59 ± 2.43	0.05 (− 0.10 to 0.20)
Vitamin E	6.77 ± 4.29	6.81 ± 4.09	− 0.01 (− 0.16 to 0.14)
Vitamin C	180.27 ± 126.88	182.51 ± 129.62	− 0.02 (− 0.16 to 0.13)
Thiamine	1.18 ± 0.61	1.18 ± 0.57	− 0.01 (− 0.16 to 0.14)
Riboflavin	1.58 ± 0.80	1.58 ± 0.70	− 0.001 (− 0.15 to 0.15)
Niacin	15.51 ± 10.20	17.57 ± 7.43	0.09 (− 0.05 to 0.24)
Vitamin B_6_	1.40 ± 0.84	1.40 ± 0.76	0.01 (− 0.14 to 0.16)
Vitamin B_12_	4.30 ± 5.67	4.38 ± 4.04	− 0.01 (− 0.16 to 0.13)
Folate	334.17 ± 171.48	337.61 ± 159.09	− 0.02 (− 0.17 to 0.13)
Anthocyanins	71.83 ± 56.49	64.85 ± 56.35	0.12 (− 0.02 to 0.27)
Chalcons	0.01 ± 0.01	0.01 ± 0.01	− 0.14 (− 0.29 to 0.003)
Dihydrocalcones	4.08 ± 4.57	4.06 ± 4.90	0.004 (− 0.14 to 0.15)
Dihydroflavonols	8.22 ± 10.75	5.45 ± 8.21	0.26 (0.12 to 0.41)
Flavanols	101.36 ± 68.14	97.09 ± 74.97	0.06 (− 0.09 to 0.21)
Flavanones	56.27 ± 57.19	52.92 ± 53.57	0.06 (− 0.09 to 0.21)
Flavones	14.42 ± 11.03	15.10 ± 13.00	− 0.06 (− 0.21 to 0.09)
Flavonols	35.01 ± 30.34	34.84 ± 31.38	0.006 (− 0.14 to 0.15)
Isoflavonoids	0.0003 ± 0.001	0.0002 ± 0.001	0.11 (− 0.04 to 0.26)
Hydroxybenzoic acids	26.85 ± 25.22	25.88 ± 22.95	0.04 (− 0.11 to 0.19)
Hydroxycinnamic acids	183.20 ± 99.67	177.62 ± 103.91	0.05 (− 0.09 to 0.20)
Hydroxyphenylacetic acids	1.01 ± 1.72	0.85 ± 1.48	0.09 (− 0.05 to 0.24)
Hydroxyphenylpropanoic acids	0.44 ± 0.89	0.38 ± 0.76	0.06 (− 0.09 to 0.21)
Stilbeni	4.90 ± 6.21	3.28 ± 4.78	0.27 (0.12 to 0.42)
Lignans	10.78 ± 9.64	10.37 ± 9.16	0.04 (− 0.11 to 0.19)
Achylmethoxyphenols	0.03 ± 0.11	0.02 ± 0.08	0.11 (− 0.04 to 0.26)
Achylphenols	1.65 ± 1.25	1.63 ± 1.20	0.02 (− 0.13 to 0.17)
Furanocoumarins	1.13 ± 1.69	0.99 ± 1.45	0.08 (− 0.06 to 0.23)
Hydroxybenzaldehydes	0.52 ± 0.67	0.34 ± 0.51	0.27 (0.12 to 0.42)
Hydroxybenzoketones	0.001 ± 0.002	0.0004 ± 0.002	0.11 (− 0.04 to 0.26)
Hydroxycoumarins	0.43 ± 0.54	0.28 ± 0.41	0.27 (0.12 to 0.42)
Naphthoquinones	0.01 ± 0.03	0.004 ± 0.01	0.17 (0.02 to 0.31)
Tyrosols	17.57 ± 31.48	14.95 ± 27.11	0.08 (− 0.06 to 0.23)

The Salus in Apulia study (*n* = *1502*)

*BMI* body mass index, *ARHL* age-related hearing loss, *COPD* chronic obstructive pulmonary disease, *MMSE* mini-mental state examination, *LOD* late-onset depression, *EOD* early-onset depression, *HDL* high-density lipoprotein, *LDL* low-density lipoprotein, *RBC* red blood cell count, *WBC* white blood cell, *HbA1* glycated hemoglobin, *AST* aspartate transaminase, *ALT* alanine aminotransferase, *GGT* γ-glutamil transferasi, *IL-6* interleukin-6, *TNF-α* tumor growth factor-α, *PCR* C-reactive protein, *APOE* apolipoprotein E, *EPA* eicosapentaenoic acid, *DHA* docosahexaenoic acid

*As Mean and Standard Deviation for continuous variable, percentage (%) for categorical

^ψ^Hedges’s effect size; (95% CI), Confidential Interval at 95% food groups and nutrients were calculated on quantity daily consumption

^¥^Symbol represents specify how micronutrients were calculated

Table 2 shows LASSO regression outputs for variable selection across food groups, macronutrients, and micronutrients, and their corresponding coefficients for different penalty parameter values (*λ*). At *λ* = 0.012, only five non-zero food groups remained in the model: legumes, caloric drinks, coffee, wine, spirits, and water (coefficient of variation (CV) mean deviance: 0.782). When *λ* approached 0.011, only the micronutrients calcium, zinc, flavanones, furanocoumarins, hydroxycoumarins, and naphthoquinones conferred the largest signal in the model (CV mean deviance: 0.007), while when *λ* approached 0.003, only water, carbohydrate soluble, cholesterol, palmitic acid, stearic acid, polyunsaturated fatty acids (PUFA), docosahexaenoic acid (DHA), and alcohol were selected as the best predictors in the model (CV mean deviance: 0.809).

**Table 2:** Lasso regression for selection variable for physical frailty as outcome on food groups and macronutrients

Parameters*	*λ*	CV mean deviance
Food groups	0.012	0.782
Legumes		
Caloric drinks		
Coffee		
Wine		
Spirits		
Water		
Macronutrients	0.003	0.853
H_2_O		
Carbohydrates soluble		
Cholesterol		
Palmitic acid		
Stearic acid		
Polyunsaturated fatty acid		
DHA		
Alcohol		
Micronutrients	0.011	0.007
Ca		
Zn		
Flavanones		
Furanocoumarins		
Hydroxycoumarins		
Naphthoquinones		

*λ Lambda selected by cross-validation

The above dietary variables, found to be potentially most influential on the physical frailty condition, were further fitted into both raw and adjusted logistic regression models performed for each of the three clusters of variables (food groups, macronutrients, and micronutrients) to evaluate the direction and weight of each one on the physical frailty odds risk, as shown in Table 3. Higher consumption of coffee, wine, and spirits was found to be inversely associated to physical frailty outcome (OR 0.992, 95% CI 0.987–0.997, OR 0.998, 95% CI 0.997–0.999, and OR 0.940, 95% CI 0.891–0.9934 respectively) in raw models, while only wine (OR 0.998, 95% CI 0.997–0.999) and coffee (OR 0.998, 95% CI 0.997–0.999) showed signs of association after controlling for major confounders, i.e., age, sex, education, depression, cognitive impairment, diabetes, and obesity. By contrast, legumes were directly associated with physical frailty in both raw and adjusted models (OR 1.005, 95% CI 1.000–1.009). Notwithstanding, the closeness to 1 of the ORs for these foods across the logistics leaves room for inference of small association effects, presumably explained by the large sample size.

**Table 3:** Logistic regression of physical frailty on food groups, macro-, and micronutrients, together in the model

Parameters^*^	Not adjusted				Adjusted^§^			
	OR	Se (OR)	*p*	CI (95%)	OR	Se (OR)	*p*	CI (95%)
Foodgroups								
Legumes	1.005	0.002	0.016	1.001 to 1.009	1.005	0.002	0.019	1.000 to 1.009
Caloric drinks	0.998	0.002	0.422	0.994 to 1.002	0.998	0.002	0.451	0.994 to 1.002
Coffee	0.992	0.003	0.004	0.987 to 0.997	0.994	0.003	0.033	0.989 to 0.999
Wine	0.998	0.001	0.001	0.997 to 0.999	0.998	0.001	0.004	0.997 to 0.999
Spirits	0.940	0.026	0.028	0.891 to 0.9934	0.951	0.025	0.063	0.928 to 1.022
Water	1.000	0.0002	0.067	0.999 to 1.001	1.000	0.0002	0.073	0.999 to 1.001
Age	–	–	–	–	1.027	0.013	0.030	1.002 to 1.052
Gender	–	–	–	–	1.135	0.190	0.450	0.858 to 1.667
Education	–	–	–	–	0.977	0.022	0.299	0.935 to 1.021
Depression	–	–	–	–	0.905	0.219	0.681	0.564 to 1.454
Cognitive impairment	–	–	–	–	1.068	0.392	0.857	0.521 to 2.192
Diabetes	–	–	–	–	1.713	0.351	0.009	1.147 to 2.560
Obesity	–	–	–	–	1.310	0.211	0.094	0.955 to 1.797
Macronutrients								
H_2_O	1.000	0.0002	0.075	0.999 to 1.001	1.000	0.0002	0.063	0.999 to 1.001
Carbohydrates soluble	0.996	0.002	0.166	0.991 to 1.001	0.996	0.002	0.144	0.992 to 1.002
Cholesterol	0.999	0.001	0.400	0.995 to 1.001	0.999	0.001	0.382	0.996 to 1.002
Palmitic acid	1.007	0.038	0.860	0.935 to 1.084	1.007	0.037	0.852	0.932 to 1.079
Stearic acid	1.209	0.165	0.166	0.924 to .581	1.190	0.163	0.204	0.906 to 1.549
Polyunsaturated fatty acid	0.924	0.023	0.002	0.880 to 0.971	0.939	0.024	0.015	0.896 to 0.991
DHA	0.624	0.416	0.480	0.169 to 2.308	0.722	0.479	0.624	0.186 to 2.559
Alcohol	0.978	0.005	< 0.001	0.967 to 0.989	0.980	0.006	0.001	0.969 to 0.992
Age	–	–	–	–	1.028	0.013	0.029	1.003 to 1.054
Gender	–	–	–	–	1.172	0.201	0.353	0.838 to 1.640
Education	–	–	–	–	0.976	0.022	0.273	0.934 to 1.019
Depression	–	–	–	–	0.914	0.222	0.713	0.567 to 1.471
Cognitive impairment	–	–	–	–	1.007	0.369	0.984	0.491 to 2.067
Diabetes	–	–	–	–	1.574	0.324	0.028	1.051 to 2.356
Obesity	–	–	–	–	1.307	0.211	0.098	0.952 to 1.795
Micronutrients								
Ca	1.001	0.0002	< 0.001	1.000 to 1.001	1.000	0.0002	0.001	1.000 to 1.001
Zn	0.971	0.011	0.012	0.949 to 0.993	0.977	0.012	0.048	0.952 to 0.998
Flavanones	0.999	0.001	0.523	0.996 to 1.002	0.999	0.001	0.678	0.997 to 1.002
Furanocoumarins	0.937	0.051	0.231	0.841 to 1.042	0.942	0.052	0.279	0.940 to 1.042
Hydroxycoumarns	0.605	0.118	0.010	0.412 to 0.888	0.631	0.131	0.027	0.431 to 0.971
Naphthoquinones	0.003	0.015	0.239	1.99e−07 to 46.769	0.004	0.018	0.243	3.65e−07 to 40.024
Age	–	–	–	–	1.025	0.013	0.052	0.999 to 1.051
Gender	–	–	–	–	1.162	0.2000	0.381	0.831 to 1.625
Education	–	–	–	–	0.973	0.022	0.218	0.931 to 1.016
Depression	–	–	–	–	0.914	0.220	0.708	0.569 to 1.466
Cognitive impairment	–	–	–	–	0.971	0.355	0.936	0.474 to 1.988
Diabetes	–	–	–	–	1.737	0.353	0.007	1.165 to 2.588
Obesity	–	–	–	–	1.325	0.213	0.081	0.965 to 1.817

^*^OR Odds Ratio, *SE* *(OR)* Standard Error of OR, *CI* *(95%)* Confidential Interval at 95%

^§^Adjusted for: age, gender, education, depression, cognitive impairment, diabetes, and obesity

When running the same models on macronutrients, PUFAs (OR 0.924, 95% CI 0.880–0.971 and OR 0.939, 95% CI 0.896–0.991 in the raw and adjusted model) and alcohol (OR 0.978, 95% CI 0.967–0.989 and OR 0.980, 95% CI 0.969–0.992 in the raw and adjusted model, respectively) also showed an inverse association with physical frailty. As micronutrients, zinc (OR 0.971, 95% CI 0.949–0.993 and OR 0.977, 95% CI 0.952–0.998 in the raw and adjusted model, respectively) and hydroxycoumarins (OR 0.605, 95% CI 0.412–0.888 and OR 0.631, 95% CI 0.431–0.971 in the raw and adjusted model, respectively) followed the same direction, versus a slightly opposite direction for calcium (OR 1.001, 95% CI 1.000–1.001 and OR 1.000, 95% CI 1.000–1.001 in the raw and adjusted model, respectively).

## Discussion

The present study cross-sectionally investigated the eating habits of the older population (65 +) belonging to the Salus in Apulia Mediterranean-based population to profile diet-related concerns associated with physical frailty. For this purpose, a LASSO logistic regression analysis was chosen both to avoid multicollinearity among dietary variables and to better assess the interaction between diet, as expressed by a cluster of food groups, macronutrients, and micronutrients, and the physical frailty outcome. Key findings indicated that a lower consumption of wine and coffee, as well as a cluster of macro and micronutrients led by PUFAs, zinc, and coumarins, as well as a higher legumes intake, were linked to physical frailty, regardless of age, sex, and education level. Substantiating the internal validity of our data, frail subjects were clinically profiled as having a greater burden of multimorbidity than non-frail, with higher rates of ARHL, cognitive impairment, and diabetes [5]. This is in no way surprising bearing in mind the physiological pathways of aging, that involve an insidious functional decline of multiple systems, leading to interconnected and accumulated frailty phenotypes, including sensorial, cognitive, and psychological/depressive [44, 45]. The female predominance and poor education level corroborated previous findings on the same aging phenotype [5]. In fact, the educational background of the population under study reflected a rural Mediterranean population setting, where most people attended school only for a few years and worked lifelong within the agricultural sector or small enterprises.

The higher intake of legumes reflecting our frail population profile can be jointly framed from a cultural and bromatological perspective. Indeed, especially for older individuals, either cultural, income, or even oral health reasons drive the habit of preferring legumes to animal protein sources in this area [33]; this implies both a lower dietary content of noble proteins and a certain intake of antinutrients (e.g., phytates), which act against the absorption of some micronutrients such as iron and zinc [46]. On this aspect, considering Italy from the income standpoint, the preference toward vegetable and animal proteins could decline depending on the geographical region; in southern Italy, for example, people are more adherent to a Mediterranean diet model that places high consumption of vegetables, fruits, legumes, and unprocessed cereals in the first place, but moderate consumption of fish and meat compared to people in the north. In light of this, while assuming the Mediterranean model as a whole to be healthy [47], attention must be paid to declinations not always profitable in preserving the physical well-being of the elderly.

As for beverages, the findings on coffee and wine may be understood chiefly from a bromatological but also social standpoint. First, a shared plant-based nature itself is responsible for providing many micronutrients, including antioxidants, polyphenols, and other beneficial bioactive plant compounds. In particular, the Mediterranean diet setting of our population meant an intrinsically greater exposure to plant sources such as fruits, vegetables, grains, nuts, and olive oil [33].

Findings on coffee consumption appear to be very sensitive since it is one of the most widely consumed beverages globally and currently the most consumed by Italians, whether as espresso or moka cups. Its phytochemistry is well-known to include bioactive and antioxidant components, especially phenol compounds generated by Maillard reactions during roasting. These have been targeted for their potential influence on physical performance and chronic disease prevention in humans [48]. A moderate body of evidence endorses our data supporting a greater coffee consumption acting against physical frailty outcomes. On one hand, polyphenols can promote autophagy in the liver, muscle, and heart tissue, which is critical for renewing mitochondria, preventing mitochondrial damage during physical activity, and improving and maintaining muscle mass and endurance. On the other, coffee may improve insulin sensitivity and glucose uptake into muscle, thus allowing better trophism [49]. The little body of longitudinal evidence supports the plausibility that coffee may indirectly reduce the risk of physical disabilities, including frailty, by slowing age-related sarcopenia and muscle wasting [50]. The same report claimed that a moderate daily amount of coffee might curb the onset of chronic diseases such as diabetes, cardiovascular disease, and Alzheimer’s disease, all known contributors to a declining physical function during aging [51].

As to our findings about wine, our results showed an inverse association with physical frailty and even alcohol, as considered apart in further pooled analyses. From an etiopathological viewpoint, a high alcohol consumption is widely reported to exacerbate the accumulation paths of chronic illnesses by primarily affecting the liver, and we recently documented how liver damage shortens the lifespan of frail individuals [52]. However, we have to translate this finding from a social perspective, as wine (and coffee too) are both beverages enjoyed in convivial settings [53], to which frail individuals are rarely accustomed [45]. Indeed, a moderate alcohol consumption might facilitate social bonding [54], helping to build or strengthen social support or networks and thus prevent social isolation [55]. A body of literature has consistently claimed that the social domain is embodied in some multidimensional fragility concepts [56]. However, alcohol consumption on physical functioning has also gained some positive evidence, though this is still somewhat controversial. On this point, a very recent meta-analysis provided the first pooled evidence that a higher alcohol consumption is associated with lower incident frailty than non-drinking among community-dwelling aging populations [57]. Consistently, a recent longitudinal survey by Kojima and colleagues providing data on alcohol consumption and the risk of incident frailty concluded that non-drinkers are more likely to develop frailty than those with low alcohol consumption, but leaving some explanation in the poorer baseline health status [58].

Moreover, when considering the alcohol issue in a matrix context, meaning the beverage as a whole, the nutritional value of wine should be pointed out; its rich content of polyphenols is renowned for being effective in preventing chronic diseases because of the antioxidant and anti-inflammatory effect of compounds such as resveratrol, and non-flavonoid phenols, such as stilbenes. On this front, the one longitudinal report on humans reported an association of high long-term exposure to dietary resveratrol with a lower risk of developing frailty in older adults over a 3-year follow-up [59]. A possible explanation could be sought in resveratrol’s ability to interact with SIRT1 in inhibiting inflammatory and apoptotic signals and thus slowing down aging skeletal muscle mass deterioration.

Among wine polyphenols, the micronutrient coumarins was found to retain significance as inversely associated with the physical frailty status in adjusted logistic models. In this respect, it is known that higher levels of coumarins are typically found in red wines that have aged longer in newer barrels. Their antioxidant capacity has been described as the direct scavenging of reactive oxygen and nitrogen species (ROS) and other mechanisms such as metal chelation [60]. However, the multiple reported bioactive anticoagulant, anti-inflammatory, anticancer, and enzyme inhibition properties do not exclude the possibility that coumarins may also be implicated in processes that could trigger the onset of frailty [61].

Then, a lower intake of PUFAs and zinc was also found to be associated to frailty in further clustering analyses. Here, the biological explanation behind the unsaturated fatty acid pattern, including essential n−3 and n−6, with respect to a better physical state, may rely on the anti-inflammatory properties of their derivatives. Indeed, age-related inflammation can lead to muscle wasting and thus contribute to sarcopenia and deteriorating gait speed. The ability of PUFAs to increase the muscle protein anabolic response to insulin and stimulate muscle protein synthesis has been well-established in both animals and humans [62, 63]. Recently, serum levels of n−3 PUFAs have been suggested as a marker for frailty risk, since a lower concentration of eicosapentaenoic and docosahexaenoic acid was detected in human erythrocytes [64].

As for zinc, of which a lower daily consumption was equally found to be associated to frailty, evidence regarding the immune function, bone mass, cognitive function, and oxidative stress [65, 66] makes it an essential micronutrient in aging. Lean meats and seafood are good sources of zinc, followed by grains and other plant sources such as nuts. Some reports have also pointed to a biological role of zinc as an appetite stimulator in the regulation of food intake via hypothalamus paths, and suggestions about its clinical application in anorexia nervosa, cachexia, and sarcopenia are not new [67–69]. Importantly, our borderline findings regarding a possible role for calcium in frailty settings open a window for debate. In this respect, a team of experts recently conducted analyses of calcium associated with age, mortality, and clinical frailty in three different cohort studies on aging and their demographic subsets. The authors considered highly heterogeneous reports, emphasizing extreme caution in generalizing this finding in the context of aging [70].

### Strengths and limitations

Strengths of this study include the fairly large sample size, the generalizability of the results to the South-Italian population, the use of a larger number of foods at the assessment of dietary habits, and the in-depth investigation of dietary habits through the use of Food Frequency Questionnaires (FFQ) enquiring a large number of foods, macronutrients, and micronutrients. Instead, limitations include risk of bias due to social desirability on food recall, and the cross-sectional design, which precludes understanding the temporal nature of the associations: hence, prospective studies are needed to clarify any causal relationship in this context. Also, the large sample size may have led to the small association effects, thus partially undermining the accuracy of findings. Lastly, the impairment of cognitive functions, particularly memory, measured by MMSE could lead to a worse recall bias when filling out the FFQ.

## Conclusions

This cross-sectional survey conducted in a Mediterranean area accustomed to eating a traditional plant-based diet suggests that a lower consumption of coffee and wine, as well as PUFAs, zinc, and coumarins, but a higher intake of legumes, are associated to a physical frailty aging profile. From a food literacy perspective in favor of healthy aging, our results suggest coffee and wine be a good food choice, yet pending causal corroboration of the claim.

## Supplementary Information

Below is the link to the electronic supplementary material.

Supplementary file1 Concordance of the single foods in the questionnaire and the food grouping used in the analyses (DOCX 32 KB)

## Data Availability

Raw data can be provided upon email request.
