# Correction to: Dietary profiling of physical frailty in older age phenotypes using a machine learning approach: the Salus in Apulia Study

**DOI:** 10.1007/s00394-023-03174-0

**Published:** 2023-05-17

**Authors:** Sara De Nucci, Roberta Zupo, Rossella Donghia, Fabio Castellana, Domenico Lofù, Simona Aresta, Vito Guerra, Ilaria Bortone, Luisa Lampignano, Giovanni De Pergola, Madia Lozupone, Rossella Tatoli, Giancarlo Sborgia, Sarah Tirelli, Francesco Panza, Tommaso Di Noia, Rodolfo Sardone

**Affiliations:** 1 Unit of Data Sciences and Technology Innovation for Population Health, National Institute of Gastroenterology-IRCCS “Saverio de Bellis”, Castellana Grotte, Bari, Italy; 2 Department of Electrical and Information Engineering, Polytechnic of Bari, Bari, Italy; 3 Unit of Geriatrics and Internal Medicine, National Institute of Gastroenterology-IRCCS “Saverio de Bellis,” Research Hospital, Castellana Grotte, Bari, Italy; 4 Department of Basic Medical Sciences, Neuroscience and Sense Organs, University of Bari “Aldo Moro”, Bari, Italy

**Correction to: European Journal of Nutrition (2023) 62:1217–1229** 10.1007/s00394-022-03066-9

The original version of this article unfortunately contained a mistake. Affiliations 1 and 3 were incorrectly given as

^1^Unit of Data Sciences and Technology Innovation for Population Health, National Institute of Gastroenterology “Saverio de Bellis,” Research Hospital, Castellana Grotte, Bari, Italy

^3^Unit of Geriatrics and Internal Medicine, National Institute of Gastroenterology “Saverio de Bellis,” Research Hospital, Castellana Grotte, Bari, Italy

but should have been

^1^Unit of Data Sciences and Technology Innovation for Population Health, National Institute of Gastroenterology-IRCCS “Saverio de Bellis”, Castellana Grotte, Bari, Italy

^3^Unit of Geriatrics and Internal Medicine, National Institute of Gastroenterology-IRCCS “Saverio de Bellis,” Research Hospital, Castellana Grotte, Bari, Italy.

