# Supplementary material for: Dietary profiling of physical frailty in older age phenotypes using a machine learning approach: the Salus in Apulia Study
Source: Eur J Nutr. 2022 Dec 9;62(3):1217–29. doi: 10.1007/s00394-022-03066-9 (PMC10030526; doi:10.1007/s00394-022-03066-9)
Supplement: Supplementary file 1 — Supplementary file1 Concordance of the single foods in the questionnaire and the food grouping used in the analyses (DOCX 32 KB) [file 394_2022_3066_MOESM1_ESM.docx]

**Supplementary Table S1. Concordance of the single foods in the questionnaire and the food grouping used in the analyses**

Original Article

**Title:** Dietary Profiling of Physical Frailty in Older Age Phenotypes Using a Machine Learning Approach: The Salus in Apulia Study

**Authors:** Sara De Nucci^1^, Roberta Zupo^1^**†**, Rossella Donghia^1^, Fabio Castellana^1^, Domenico Lofù^2^, Simona Aresta^2^, Vito Guerra^1^, Ilaria Bortone^1^, Luisa Lampignano^1^, Giovanni De Pergola^3^, Madia Lozupone^1^, Rossella Tatoli^1^, Giancarlo Sborgia^4^, Sarah Tirelli^1^, Gianluigi Giannelli^5^, Francesco Panza^1^, Tommaso Di Noia^2^ and Rodolfo Sardone^1^

^1^ Unit of Data Sciences and Technology Innovation for Population Health, National Institute of Gastroenterology “Saverio de Bellis,” Research Hospital, Castellana Grotte, Bari, Italy

^2^ Department of Electrical and Information Engineering, Polytechnic of Bari, Bari, Italy

^3^ Unit of Geriatrics and Internal Medicine, National Institute of Gastroenterology “Saverio de Bellis,” Research Hospital, Castellana Grotte, Bari, Italy

^4^ Department of Basic Medical Sciences, Neuroscience and Sense Organs, University of Bari “Aldo Moro”, Bari, Italy

^5^Scientific Direction, National Institute of Gastroenterology "Saverio de Bellis", Research Hospital, Castellana Grotte, Bari, Italy

**†Corresponding author:** Roberta Zupo, MSc

|  | **Final food groups** | **Single foods from questionnaire** |  |
| --- | --- | --- | --- |
| 1 | DAIRY | Latte intero (*whole-fat milk*), Scamorza-Caciottina fresca-Stracchino-Fontina (semi-seasoned italian cheese), Bel Paese-Gorgonzola (italian blue cheese), Provolone-Caciocavallo (seasoned italian cheese), Grana-Parmigiano, Svizzero (*swiss cheese*), Pecorino-Vacchino (*goat cheese, cow cheese*), Formaggino, (*cheese spread*), Mozzarella (*mozzarella cheese*), Gelato (*ice cream*), Yogurt |  |
| 2 | LOW FAT DAIRY | Latte scremato – parzialmente scremato (*skimmed and semi-skimmed milk*), Ricotta (*cottage cheese*) |  |
| 3 | EGGS | Uova (*eggs)* |  |
| 4 | WHITE MEAT | Pollo (*chicken*), Coniglio (*rabbit*) |  |
| 5 | RED MEAT | Vitello (*veal*), Cavallo (*horse*), Maiale (*Pork*), Fegato (*liver*), Agnello (*lamb*) |  |
| 6 | PROCESSED MEAT | Salsiccia fresca (*fresh sausages*), Prosciutto crudo (*raw ham*), Mortadella (a tipical italian cured meat),Prosciutto cotto (ham), Salame (*salami*) |  |
| 7 | FISH | Sogliola-Orata-Dentice-Spigola-Cernia (*sole, sea bream, snapper, sea bass, grouper*), Merluzzo-Razza-Palombo (*codfish, stingray, dogfish*), Triglia-Cefalo-Sgombro (*goatfish, mullet, mackerel*), Acciughe-Sarde (*anchovies, sardines*), Tonno sott’olio (*tuna in oil*) |  |
| 8 | SEAFOOD/SHELLFISH | Polpo-Seppie-Calamari-Gamberi (*octopus, cuttlefish, squid, prawns)*, Cozze-Altri frutti di mare (*mussels, other seafoods*) |  |
| 9 | LEAFY VEGETABLES | Spinaci (*spinach*), Bietole-Cicorie (*chard, chicory*), Insalata (*salad*) |  |
| 10 | FRUITING VEGETABLES | Pomodori (*tomatoes*), Zucchine-Melanzane (*zucchini, eggplants*), Peperoni (*peppers*), Carciofi (*artichokes*) Cetrioli-cocomeri (*cucumbers*) |  |
| 11 | ROOT VEGETABLES | Carote (*carrots*) |  |
| 12 | OTHER VEGETABLES | Minestrone (*vegetable soup*), Cavoli–Cavolfiori-Cime di Rape-Rape (*cabbage, cauliflower, broccoli, green turnips*), Finocchi- Sedano (*fennels, celery)* |  |
| 13 | LEGUMES | Ceci - Lenticchie – Fagioli (*chickpeas, lentils, beans*), Piselli (*peas*), Fagiolini (*green beans*), Fave con Verdura (*broad beans with vegetables*) |  |
| 14 | POTATOES | Patate (*potatoes*) |  |
| 15 | FRUITS | Arance-Mandarini-Pompelmi (*oranges, tangerines, grapefruits*), Pesche (*peaches*), Fichi (*figs*), Albicocche (*apricots*), Uva (*grapes*), Anguria (*watermelon*), Melone giallo (*melon*), Mele-Pere (*apples, pears*), Kiwi, Ciliege (cherries), Banane |  |
| 16 | NUTS | Frutta secca (*nuts*) |  |
| 17 | GRAINS | Pane (*bread*), Pasta asciutta (*pasta*), Riso o risotti (*rice or risotti*), Pastina o riso in brodo (*pasta or rice in broth*) |  |
| 18 | OLIVES AND VEGETABLE OIL | Olive da tavola (*olives*), Olio di oliva (*olive oil*) |  |
| 19 | COOKING  EDIBLE FATS | Olio di semi (*seed oil*), Olio di oliva per frittura (*olive oil for frying*), Olio di semi per frittura (*seeds oil for frying*), Olio di oliva per cucinare (*olive oil for cooking*), Olio di semi per cucinare (*seeds oil for cooking*), Burro (*butter*), Margarina (*margarine*), Burro per frittura (*butter for frying*), Margarina per frittura (*margarine for frying*), Burro per cucinare (*butter for cooking*), Margarina per cucinare (*margarine for cooking*) |  |
| 20 | SWEETS | Caramelle (*sweets*), Cioccolata (*chocolate*), Pasticceria (*pastries*), Biscotti – Paste secche (*cookies, biscuits*) |  |
| 21 | SUGARY | Zucchero (*sugar*), Frutta sciroppata (*fruit in syrup*) |  |
| 22 | JUICES | Succhi di frutta (*fruit juice*) |  |
| 23 | CALORIC DRINKS | Coca Cola – Aranciata – Chinotto (*coke, orange juice, chinotto*) |  |
| 24 | READY TO EAT DISH | Pizza, Focaccia (a tipical Apulian bakery product) |  |
| 25 | COFFEE | Caffè (*coffee*), Caffè d’orzo (*barley coffee*) |  |
| 26 | WINE | Vino (*wine*) |  |
| 27 | BEER | Birra (*beer*) |  |
| 28 | SPIRITS | Liquore (*liquor*) |  |
| 29 | WATER | Acqua (*water*) |  |
